# Checkpoint inhibition in combination with an immunoboost of external beam radiotherapy in solid tumors (CHEERS): study protocol for a phase 2, open-label, randomized controlled trial

**DOI:** 10.1186/s12885-021-08088-w

**Published:** 2021-05-07

**Authors:** Mathieu Spaas, Nora Sundahl, Eva Hulstaert, Vibeke Kruse, Sylvie Rottey, Daan De Maeseneer, Veerle Surmont, Annabel Meireson, Lieve Brochez, Dries Reynders, Els Goetghebeur, Robbe Van den Begin, Dirk Van Gestel, Vincent Renard, Piet Dirix, Pieter Mestdagh, Piet Ost

**Affiliations:** 1 Radiation Oncology, Ghent University Hospital, C. Heymanslaan 10, B-9000 Ghent, Belgium; 2 Dermatology, Ghent University Hospital, Ghent, Belgium; 3 Center for Medical Genetics (CMGG), Ghent University, Ghent, Belgium; 4 Cancer Research Institute Ghent (CRIG), Ghent University, Ghent, Belgium; 5 Medical Oncology, Ghent University Hospital, Ghent, Belgium; 6 Medical Oncology, AZ Sint-Lucas, Bruges, Belgium; 7 Pulmonary Medicine, Ghent University Hospital, Ghent, Belgium; 8 Department of Applied Mathematics, Computer Science and Statistics & Stat-Gent CRESCENDO Consortium, Ghent University, Ghent, Belgium; 9 Radiation Oncology, Jules Bordet Insitute, Université Libre de Bruxelles, Brussels, Belgium; 10 Medical Oncology, AZ Sint-Lucas, Ghent, Belgium; 11 Radiation Oncology, Iridium Cancer Network, Wilrijk, Belgium

**Keywords:** Head and neck squamous cell carcinoma, Melanoma, Non-small-cell lung carcinoma, Renal cell carcinoma, Transitional cell carcinoma, Immunotherapy, Checkpoint inhibitor, Stereotactic body radiotherapy, Survival, Clinical trial

## Abstract

**Background:**

While the introduction of checkpoint inhibitors (CPIs) as standard of care treatment for various tumor types has led to considerable improvements in clinical outcome, the majority of patients still fail to respond. Preclinical data suggest that stereotactic body radiotherapy (SBRT) could work synergistically with CPIs by acting as an in situ cancer vaccine, thus potentially increasing response rates and prolonging disease control. Though SBRT administered concurrently with CPIs has been shown to be safe, evidence of its efficacy from large randomized trials is still lacking. The aim of this multicenter randomized phase II trial is to assess whether SBRT administered concurrently with CPIs could prolong progression-free survival as compared to standard of care in patients with advanced solid tumors.

**Methods/design:**

Ninety-eight patients with locally advanced or metastatic disease will be randomized in a 1:1 fashion to receive CPI treatment combined with SBRT (Arm A) or CPI monotherapy (Arm B). Randomization will be stratified according to tumor histology (melanoma, renal, urothelial, head and neck squamous cell or non-small cell lung carcinoma) and disease burden (≤ or > 3 cancer lesions). The recommended SBRT dose is 24Gy in 3 fractions, which will be administered to a maximum of 3 lesions and is to be completed prior to the second or third CPI cycle (depending on CPI treatment schedule). The study’s primary endpoint is progression-free survival as per iRECIST. Secondary endpoints include overall survival, objective response, local control, quality of life and toxicity. Translational analyses will be performed using blood, fecal and tissue samples. *Discussion:* The CHEERS trial will provide further insights into the clinical and immunological impact of SBRT when combined with CPIs in patients with advanced solid tumors. Furthermore, study results will inform the design of future immuno-radiotherapy trials.

**Trial registration:**

Clinicaltrials.gov identifier: NCT03511391. Registered 17 April 2018.

**Supplementary Information:**

The online version contains supplementary material available at 10.1186/s12885-021-08088-w.

## Background

Recently, checkpoint inhibitors (CPIs) have become the standard of care (SOC) as a first or second line systemic treatment for patients with inoperable or metastatic non-small cell lung cancer, renal cell cancer, urothelial cancer, melanoma and head and neck cancer [1–4]. Unfortunately, the majority of patients do not respond to this treatment and the overall survival (OS) remains limited. Patients who do not respond to CPIs typically have less immunogenic tumors with low levels of tumor-infiltrating cluster of differentiation 8 positive (CD8+) T cells [5, 6]. It is possible that in these non-responding patients, the tumor microenvironment hinders T-cell infiltration and induction of an endogenous immune response.

Pre-clinical and clinical evidence indicate that radiotherapy can act as an in-situ cancer vaccine by inducing immunogenic cell death, triggering the release of tumor-derived antigens and attracting CD8+ T cells to the tumor and hereby leading to an increase in immunogenicity. This can in turn elicit a – potentially systemic – anti-tumor immune response [7–10], which may lead to tumor responses outside the irradiated regions, a phenomenon called the “abscopal” effect, as previously reported in several kinds of malignancies [11–15]. Pre-clinical evidence indicates that hypofractionated radiotherapy (e.g. 3 × 8 Gy) might be best suited to trigger systemic immune effects [16]. Stereotactic body radiotherapy (SBRT) is an innovative technique which allows the safe administration of this hypofractionated radiotherapy with high precision and limited toxicity.

The combination of CPIs with SBRT could therefore work synergistically and lead to an improved systemic immune response, with higher response rates and longer OS as a result [7–9, 11]. Apart from a systemic abscopal effect, clinical evidence also indicates that the combination of radiotherapy with CPIs leads to excellent local responses of irradiated tumors, denoted as the “adscopal” effect [8].

Early phase clinical trials have demonstrated that CPIs can be combined safely with radiotherapy without excess toxicity [8, 9]. In our center, we have conducted 2 phase I trials and 1 phase II trial combining CPIs with SBRT [17–19]. We did not observe additional toxicity with the combined use of CPIs with SBRT. The current randomized trial will shed light on the efficacy of this combination treatment.

## Methods/design

**Fig. 1:**
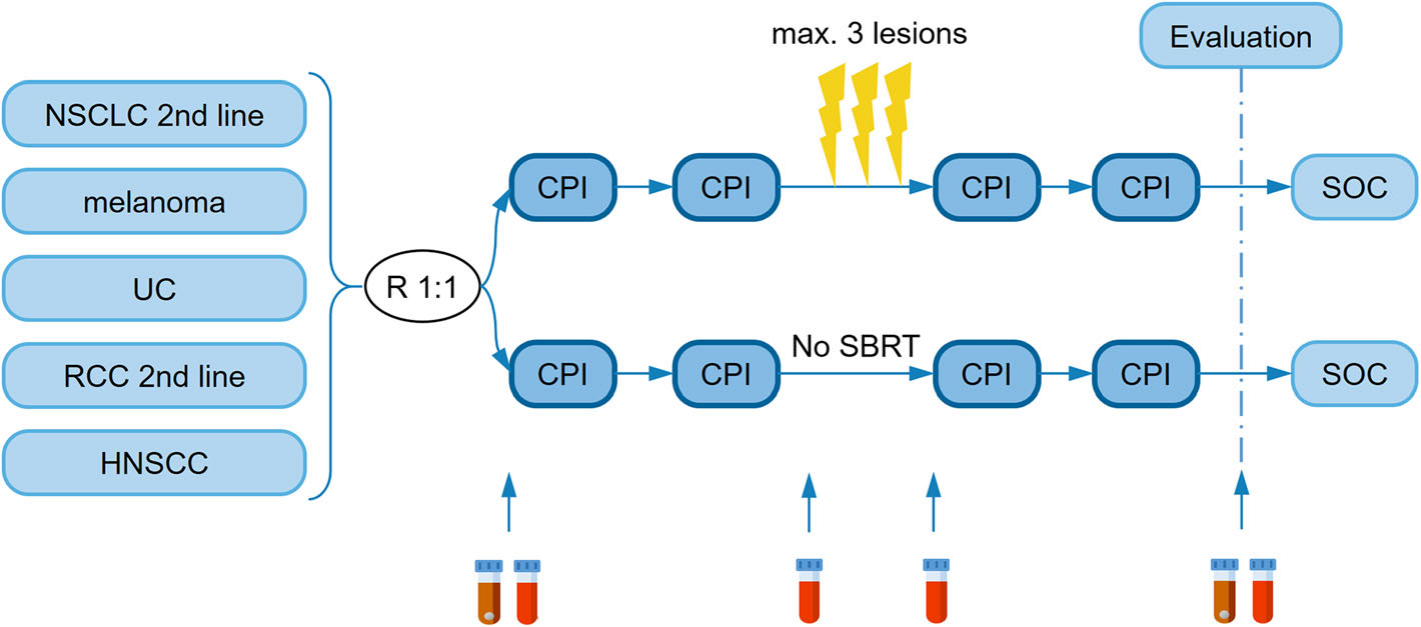
CHEERS trial design. CPI, checkpoint inhibitor; HNSCC, head and neck squamous cell carcinoma; NSCLC, non-small cell lung carcinoma; RCC, renal cell carcinoma; SBRT, stereotactic body radiotherapy; SOC, standard of care; UC, urothelial carcinoma. Brown vial, fecal sample; red vial, blood sample; lightning bolt, radiotherapy fraction

This study is approved by the Ghent University Hospital Ethics committee (EC2017/1678) and is registered on clinicaltrials.gov (NCT03511391). Patients diagnosed with locally advanced or metastatic melanoma, renal cell carcinoma, non-small cell lung carcinoma, urothelial carcinoma or head- and neck squamous cell carcinoma will be entered in a randomized phase II trial. In the interventional arm A, patients will undergo SBRT to maximally three extracranial lesions in addition to SOC CPI treatment. Patients in the control arm B will only receive SOC CPIs (Fig. 1).

### Objectives

- Primary endpoint:
  - Progression-free survival (PFS) will be analyzed in all patients who received at least one cycle of CPIs. PFS is defined as the time from randomization to disease progression or death from any cause. Disease progression will be evaluated using systemic imaging by computed tomography (CT) or positron emission tomography (PET) CT and will be defined according to immune Response Evaluation Criteria in Solid Tumors (iRECIST) [20]. Imaging will occur every 3 months, or earlier if clinically indicated.
- Secondary endpoints:
  - Overall survival (OS) is defined as the time from trial randomization to the date of death from any cause.
  - Best objective overall response as per iRECIST and RECIST 1.1 [21].
  - Local control of the irradiated lesion(s) will be assessed per RECIST 1.1.
  - Adverse events will be scored using the Common Terminology Criteria for Adverse Events (CTCAE) version 5.0.
  - Quality of life (QOL) scoring using the EORTC QLQ-C30. Raw scores will be transformed to a linear scale ranging from 0 to 100. The results will be presented in accordance with the most recent guidelines for reporting health related QOL randomized controlled trials.
  - Systemic responses during the trial will be assessed using peripheral blood samples. Using flow cytometry, subsets of immune cells, selected immune checkpoints and cell death markers will be identified. Cytokines will be measured via multiplex analysis. Cell-free DNA will be analyzed using shallow whole genome sequencing which will allow reliable non-invasive copy-number profiling, as previously demonstrated by our group [22]. In addition, germline DNA and circulating extracellular RNA will be analyzed. If at any point prior to or during the study, tissue samples of the primary tumor or metastatic foci were obtained from the trial patient for diagnostic purposes and stored, any remaining tissue may be retrieved from the respective pathology department. Analyses of this formalin-fixed paraffin embedded (FFPE) material will include isolation of tumor RNA and DNA, programmed death-ligand 1 (PD-L1) expression and tumor infiltrating lymphocytes (TIL). Depending on the published scientific literature available at the time of data cut off, additional analyses could be performed.
  - The predictive and prognostic value of the composition of the gut microbiome will be assessed using a serial fecal sampling.

#### Inclusion criteria

- Before patient registration, written informed consent must be given according to ICH/GCP and national/local regulations.
- Histologically confirmed diagnosis of a solid tumor.
- At least one extracranial tumor lesion available for radiotherapy administration.
- Patient will receive a checkpoint inhibitor per standard of care in one of the following settings (locally advanced or metastatic):
  - melanoma: 1st-3rd line nivolumab or pembrolizumab
  - renal cell carcinoma: 2nd line nivolumab
  - non-small cell lung carcinoma: 2nd or 3rd line nivolumab, atezolizumab or pembrolizumab
  - urothelial carcinoma: 1st-3rd line nivolumab, atezolizumab or pembrolizumab
  - head- and neck squamous cell carcinoma: 1st-2nd line pembrolizumab, 2nd line nivolumab
- Karnofsky Performance status > 60
- Age 18 years or older.

#### Exclusion criteria

- Prior radiotherapy preventing treatment with SBRT.
- Prior treatment with an anti-PD-(L)1 antibody.
- Has a known additional malignancy that is progressing or requires active treatment. Exceptions include basal cell carcinoma of the skin or squamous cell carcinoma of the skin that has undergone potentially curative therapy or in situ cervical cancer or prostate cancer that has undergone potentially curative therapy and with normalized PSA.
- Uncontrolled central nervous system (CNS) metastases at baseline (controlled = previously-treated CNS metastases (surgery ± radiotherapy, radiosurgery, or gamma knife) and who meet both of the following criteria: a) are asymptomatic and b) have no requirement for steroids or enzyme-inducing anticonvulsants), and/or carcinomatous meningitis.
- Any condition requiring systemic treatment with corticosteroids (> 10 mg daily prednisone equivalent) or other immunosuppressive medication within 14 days prior to the first dose of study drug. Inhaled steroids and adrenal replacement steroid doses > 10 mg daily prednisone equivalent are permitted in the absence of active autoimmune disease.
- Has a diagnosis of immunodeficiency or history of human immunodeficiency virus (HIV), Hepatitis B or Hepatitis C infection.
- Mental condition rendering the patient unable to understand the nature, scope and possible consequences of the study.
- Patient not likely to comply with the protocol; i.e. uncooperative attitude, inability to return for follow-up visits or unlikely to complete the study.
- Contraindication for radiotherapy.
- Female subjects of childbearing potential must be willing to use an adequate method of contraception for the course of the study through 120 days after the last dose of study medication.

#### Evaluation and randomization

Patients must be restaged within 4 weeks prior to randomization. The study will employ a 1:1 randomization between arm A: arm B, stratified on primary tumor histology (melanoma, renal cell carcinoma, non-small-cell carcinoma, urothelial carcinoma or head-and-neck squamous cell carcinoma) and on the number of metastases present at the time of inclusion (≤3 or > 3). Randomly permutated blocks of variable length 2 and 4 will thus be assigned to each stratum. Within each block, assignment to treatment and control is random but balanced. Every patient is assigned a treatment arm following the randomization-scheme. As this is an open-label trial, the randomization procedure and outcome will not be blinded.

#### Interventions

- Arm A: interventional arm:
  Patient receives 1/2 cycle(s) of CPI (depending on type of CPI). Prior to the second/third cycle, SBRT is administered to maximally 3 extracranial lesions in 3 fractions of 8 Gy, for a total dose of 24 Gy on each lesion. Patient receives 3–5 cycles of CPI in total prior to first evaluation (imaging + consultation).
  - Simulation:
    All patients will receive a CT in supine position with 3 mm CT slice thickness at the level of the tumor site. The planning simulation CT should cover the target and all organs at risk (OARs). A typical scan length should extend at least 10 cm superior and inferior beyond the treatment field borders. Support devices to increase patient comfort will be chosen depending on the tumor localization. Lung and liver tumor sites will be simulated with 4D-CT, taking into account breathing. The isocenter will be determined on the CT-simulator with marking of laser lines on the patient. Imaging data will be transferred to the treatment planning system. The types of OARs delineated depend on the localization of the metastasis.
  - SBRT target and OAR definition:
    Gross Target Volume (GTV): all visible tumor by combining iconographic and metabolic information (if available). No clinical target volume will be delineated.
    Planning Target Volume (PTV): expansion from the GTV to account for organ motion and setup error. Margins depend on the site irradiated with 2 mm margins for bony lesions and 5 mm for other sites. In case of overlap between OAR and PTV exists, a PTV_optim is created by subtracting the OAR or the planning organ at risk volume (PRV) from the PTV volume. This PTV_optim will be used to prescribe the dose instead of the PTV.
    A Planning Organ at Risk Volume (PRV) expansion of 2 mm will be added to the spinal cord, esophagus, intestine, etc. (if applicable), and dose constraints apply to this PRV. It is strongly recommended that dose constraints not be exceeded. If a dose constraint cannot be achieved due to overlap of the target with an OAR, the fractionation can be increased or the target coverage compromised in order to meet the constraint.
  - SBRT treatment planning and dose prescription:
    IMRT (static or rotational) treatment planning will depend on the localization of the lesion. Dose constraints of OARs will be in accordance with the recommendations from the report of the AAPM task group 101 [23]. The total dose (24 Gy = 80% of the maximal dose (30 Gy)) will be delivered in 3 fractions and fractions will be separated > 48 h and < 96 h. Treatment will be prescribed to the periphery of the target (80% of the maximal dose should cover 90% of the PTV) covering the PTV. In case of violation of dose constraints to the surrounding OARs, the prescription will be adapted accordingly.
  - SBRT delivery and verification:
    Treatment will be delivered with static or rotational IMRT with 6–18 MV photons of a linear accelerator using cone-beam CT set-up and on-line correction of patient’s position. If multiple targets will be irradiated and the targets are more than 10 cm apart in the cranio-caudal direction, multiple isocenters are needed with a CBCT prior to every treatment for every isocenter. Patient immobilization devices can be used according to the institutional policy.
- Arm B: control arm
  Patient receives 3–5 cycles of CPI with subsequent first evaluation (imaging + consultation).

#### Duration of therapy

CPI dose adjustments according to standard of care regimens are allowed after the first evaluation at the discretion of the investigator (e.g. nivolumab q2w to q4w). Patients may discontinue protocol therapy in case of radiographic disease progression (as per iRECIST). A subject may be granted an exception to continue on treatment with confirmed radiographic progression if clinically stable or clinically improved. Patients may also discontinue protocol therapy when unacceptable toxicity is encountered, in case of intercurrent illness which would in the judgment of the investigator affect patient safety or the ability to deliver treatment; or by request of the patient.

#### Concomitant medications/vaccinations (allowed & prohibited)

Medications or vaccinations specifically prohibited in the exclusion criteria are not allowed during the ongoing trial. If there is a clinical indication for one of these or other medications or vaccinations specifically prohibited during the trial, discontinuation from trial therapy or vaccination may be required. The investigator should discuss any questions regarding this with the study coordinator. The final decision on any supportive therapy or vaccination rests with the investigator and/or the subject’s primary physician.

##### Acceptable concomitant medications

All treatments that the investigator considers necessary for a subject’s welfare may be administered at the discretion of the investigator in keeping with the community standards of medical care. All concomitant medication will be recorded on the case report form (CRF) including all prescription, over-the-counter (OTC), herbal supplements, and IV medications and fluids. If changes occur during the trial period, documentation of drug dosage, frequency, route, and date may also be included on the CRF.

All concomitant medications received within 28 days before the first dose of trial treatment and 30 days after the last dose of trial treatment should be recorded. Concomitant medications administered after 30 days after the last dose of trial treatment should be recorded for SAEs.

##### Prohibited concomitant medications

Subjects are prohibited from receiving the following therapies during the Screening and Treatment Phase (including retreatment for post-complete response relapse) of this trial:

- Antineoplastic systemic chemotherapy or biological therapy
- Immunotherapy not specified in this protocol
- Chemotherapy not specified in this protocol
- Radiation therapy, other than defined in this protocol: indications for non-study prescribed radiotherapy should always be discussed first with the study sponsor.
- Live vaccines within 30 days prior to the first dose of trial treatment and while participating in the trial. Examples of live vaccines include, but are not limited to, the following: measles, mumps, rubella, varicella/zoster, yellow fever, rabies, BCG, and typhoid vaccine.

#### Adverse events reporting requirements

Patients will be instructed by the investigator to report the occurrence of any adverse event (AE). The investigator assesses and records all AEs observed during the AE reporting period from inclusion until 4 months after randomization. Special attention should be given to AEs predefined on the AE-form. All AEs are coded with the CTCAE v5.0, and assigned a grade (from 1 = mild to 5 = death related to AE) as well as a relationship to trial treatment. Grade 0 = absent must be used for the absence of predefined AEs.

Serious adverse events (SAEs) will be reported within 24 h (working days) to the central and local Ethics Committees.

#### Study calendar

- Baseline/pre-study evaluations:
  - Documentation of the patient’s medical history and all medication
  - Registration of baseline symptoms/toxicity and QOL
  - Karnofsky Performance status
  - Clinical examination
  - Blood draw assessing at least the following elements: complete blood count (hemoglobin, white blood cell count and formula, thrombocyte count), electrolytes (sodium, potassium), renal function (creatinine, blood urea nitrogen), liver function (alanine aminotransferase, aspartate aminotransferase, gamma-glutamyltransferase, alkaline phosphatase), lactate dehydrogenase and C-reactive protein.

Baseline evaluations are to be conducted within 2 weeks prior to start of checkpoint inhibitors.

- During study:
  - Registration of symptoms before first cycle of checkpoint inhibitors, at start of radiotherapy and at end of radiotherapy (if applicable) and at least every 4 weeks until 4 months after randomization. Toxicity will be assessed using the CTCAE version 5.0.
  - QOL will be assessed at baseline, at first evaluation and every 6 months thereafter. QOL will be assessed using the QLQ-C30 questionnaire.
  - A fecal sample will be obtained prior to start of CPI treatment and at the first evaluation.
  - Blood samples (EDTA and serum) for immunological analysis will be obtained prior to start of CPIs, before first fraction (Arm A), before the second/third cycle of CPIs and at every evaluation (consultation after imaging) as long as patient receives CPI (i.e. liquid biopsy).
  - Patients will be followed up until disease progression defined as per iRECIST.
  - Off-study and follow-up evaluations
  - Once a subject experiences confirmed disease progression, the subject moves into the survival follow-up phase and should be contacted by telephone every 12 weeks to assess for survival status until death, withdrawal of consent or the end of the study, whichever occurs first.
  - Monitoring of progression and survival.

Study calendars for different CPI treatment schedules are represented in Tables 1, 2, and 3.

**Table 1:** Study calendar for nivolumab 2-weekly: SBRT prior to C3; first evaluation after C5

	STUDY PERIOD											
	Enrolment	Allocation	Post-allocation								Close-out	Long-term follow-up
**TIMEPOINT**	**Pre-study**	**D-14 to -1**	**C1D1**	**C2D1**	**C3D-6**	**C3D-4 or -3**^**a**^	**C3D-1**	**C3D1**	**C4D1**	**C5D1**	**C5D +1 to +14**	**Every evaluation**^b^
**ENROLMENT**												
Eligibility screen	X											
Informed consent	X											
Allocation		X										
**INTERVENTIONS**												
SBRT fraction (arm A)					X	X	X					
Nivolumab q2w (arm A & B)			X	X				X	X	X		
**ASSESSMENTS**												
Demographics, medical Hx, performance status	X											
Imaging (CT or PET/CT)	X										X	
Fecal sample			X								X	
Liquid biopsy			X		X^c^			X				X
Adverse events			X	X	X^c^			X	X	X	X	X^d^
Quality of life			X									X^e^

*C* cycle; *CT* computed tomography; *D* day; *Hx* history; *PET* positron emission tomography; *q2w* 2-weekly; *SBRT* stereotactic body radiotherapy

^a^ SBRT is given on Wednesday-Friday-Monday or Friday-Monday-Wednesday, depending on whether nivolumab is administered on Tuesday or Thursday, respectively

^b^ Consultation after imaging; until disease progression as per iRECIST

^c^ Only for patients in study arm A

^d^ Until 3 months after radiotherapy

^e^ At least every 6 months

**Table 2:** Study calendar for nivolumab 4-weekly: SBRT prior to C2; first evaluation after C3

	STUDY PERIOD									
	Enrolment	Allocation	Post-allocation						Close-out	Long-term follow-up
**TIMEPOINT**	**Pre-study**	**D-14 to -1**	**C1D1**	**C2D-6**	**C2D-4 or -3**^**a**^	**C2D-1**	**C2D1**	**C3D1**	**C3D +1 to +14**	**Every evaluation**^b^
**ENROLMENT**										
Eligibility screen	X									
Informed consent	X									
Allocation		X								
**INTERVENTIONS**										
SBRT fraction (arm A)				X	X	X				
Nivolumab q4w (arm A & B)			X				X	X		
**ASSESSMENTS**										
Demographics, medical Hx, performance status	X									
Imaging (CT or PET/CT)	X								X	
Fecal sample			X						X	
Liquid biopsy			X	X^c^			X			X
Adverse events			X	X^c^			X	X	X	X^d^
Quality of life			X							X^e^

*C* cycle; *CT* computed tomography; *D* day; *Hx* history; *PET* positron emission tomography; *q4w* 4-*weekly SBRT* stereotactic body radiotherapy

^a^ SBRT is given on Wednesday-Friday-Monday or Friday-Monday-Wednesday, depending on whether nivolumab is administered on Tuesday or Thursday, respectively

^b^ Consultation after imaging; until disease progression as per iRECIST

^c^ Only for patients in study arm A

^d^ Until 3 months after radiotherapy

^e^ At least every 6 months

**Table 3:** Study calendar for pembrolizumab or atezolizumab 3-weekly: SBRT prior to C3; first evaluation after C4

	STUDY PERIOD										
	Enrolment	Allocation	Post-allocation							Close-out	Long-term follow-up
**TIMEPOINT**	**Pre-study**	**D-14 to -1**	**C1D1**	**C2D1**	**C3D-6**	**C3D-4 or -3**^**a**^	**C3D-1**	**C3D1**	**C4D1**	**C4D +5 to +20**	**Every evaluation**^b^
**ENROLMENT**											
Eligibility screen	X										
Informed consent	X										
Allocation		X									
**INTERVENTIONS**											
SBRT fraction (arm A)					X	X	X				
Pembrolizumab or atezolizumab q3w (arm A & B)			X	X				X	X		
**ASSESSMENTS**											
Demographics, medical Hx, performance status	X										
Imaging (CT or PET/CT)	X									X	
Fecal sample			X							X	
Liquid biopsy			X		X^c^			X			X
Adverse events			X	X	X^c^			X	X	X	X^d^
Quality of life			X								X^e^

*C* cycle; *CT* computed tomography; *D* day; *Hx* history; *PET* positron emission tomography; *q3w* `3-weekly; *SBRT* stereotactic body radiotherapy

^a^ SBRT is given on Wednesday-Friday-Monday or Friday-Monday-Wednesday, depending on whether pembrolizumab/atezolizumab is administered on Tuesday or Thursday, respectively

^b^ Consultation after imaging; until disease progression as per iRECIST

^c^ Only for patients in study arm A

^d^ Until 3 months after radiotherapy

^e^ At least every 6 months

## Statistical analysis

### Sample size

This trial is designed to assess whether SBRT to maximum 3 lesions after 2 cycles of CPIs could prolong PFS as compared to standard of care in metastatic patients. The study has a two-sided 0.05% type I error and 80% power to detect an improvement in PFS of 3 months in the intervention group as compared to the control group using a stratified log-rank test. Median PFS for the control arm was estimated at 3.1 months. Median PFS after initiation of CPIs is approximately as follows:

- 20% metastatic melanoma (1st-3rd line): 5.2 months [1, 4, 24–26]
- 20% renal cell carcinoma (2nd line): 4.6 months [27]
- 20% non-small cell lung carcinoma (2nd-3rd line): 2.7 months [3, 28, 29]
- 25% bladder cancer (1st-3rd line): 2.2 months [2, 30]
- 15% head- and neck cancer (1st-2nd line): 2.0 months [31, 32]

We estimate to recruit 30% oligometastatic patients (defined as ≤3 metastases) and 70% non-oligometastatic (defined as > 3 metastases) in the trial. We hypothesize that an improved PFS of 3 months will be seen. We chose for a 36 month accrual time and a 12 month follow-up time. Analysis is planned 9 months after inclusion of the last patient.

Using these assumptions and including a 5% at random dropout rate, the trial requires a total of 98 patients to be randomized in to two groups. Sample size calculation was performed using R version 3.4.1.

### Data analysis and monitoring

Patients will be analyzed according to the group to which they were assigned (intention-to-treat). Descriptive statistics will be used to summarize patient characteristics per treatment group.

PFS will be defined as the time from randomization to disease progression (as per iRECIST using CT or PET/CT) or death from any cause. PFS will be compared between groups using the stratified log-rank test. Kaplan-Meier estimates of 1-year PFS and OS will be provided for each treatment group and as a post-hoc subgroup analysis based on patient characteristics described above. Median follow-up time will be derived using both complete and incomplete follow-up times. Cox proportional hazards regression will be used to provide hazard ratio estimates when assessing PFS using covariates of interests. A *p*-value of less than 0.05 will be considered statistically significant.

For the evaluation of biomarkers on one time point, differences between groups will be tested using the Mann-Whitney U test. For the evaluation of biomarkers over time, differences between groups will be tested using the Wilcoxon signed-rank test. To evaluate correlations, Spearman correlation coefficients will be calculated. A p-value of less than 0.05 will be considered statistically significant.

All statistical analyses will be performed using SPSS (SPSS Inc., Chicago, Il, USA). RNA sequencing data will be analyzed using dedicated pipelines. Further processing of the count tables will be performed in R (v.3.5.1).

In order to avoid introducing bias no interim analysis will be performed. Early trial termination may be deemed necessary in case of poor accrual. As the experimental treatment carries minimal risks, no data monitoring committee will be implemented, nor will there be a predefined stopping procedure.

### Study sites and data management

This multicenter study will be conducted at the following sites: Ghent University Hospital, GZA Hospitals Antwerp, Jules Bordet Institute Brussels, Sint-Lucas Hospital Bruges and Sint-Lucas Hospital Ghent. All participating centers have extensive experience in the treatment of solid tumors. Eligible patients will be discussed at regularly scheduled multidisciplinary team meetings. Randomization and assignment of a study-specific ID will be done by the study Sponsor.

All data will be collected using the electronic case report form. The datasets generated during the study will be stored in a non-publicly available repository. All clinical records are collected and managed using REDCap (Research Electronic Data Capture) electronic data capture tools hosted at Ghent University Hospital [33], a secure web-based application designed to support data capture for research studies. All patient data (except identifying information) are stored for 25 years. Investigators from each participating institution have access to the data of their respective patients. All data are pseudonymized and patients’ details are encoded. The study Sponsor manages the entire database.

## Discussion

The CHEERS study is the first randomized phase II trial to assess the role of SBRT to maximally 3 lesions during CPI treatment as compared to CPI monotherapy in terms of PFS in patients with locally advanced or metastatic solid tumors. Its unique design will allow the effect of SBRT in combination with CPIs to be tested in a variety of tumor types, thus having the potential to greatly increase the number of patients who are eligible to receive such treatment. While most published studies to date have objectified tumor response according to RECIST 1.1, the CHEERS trial will incorporate iRECIST for response evaluations as is recommended for cancer immunotherapy trials. Other advantages such as the inclusion of a SOC control arm, as well as the integration of translational endpoints to identify potential biomarkers, are of particular interest as they will further insights into the clinical utility and underlying immune mechanisms of SBRT-CPI combination treatment.

Nevertheless, this trial also has several important limitations. While the inclusion of different tumor types will allow for more rapid accrual and reduces the risk of premature termination due to poor enrollment, our sample size will not be large enough to compare outcomes between different tumor histologies. In addition, as the comparator in this study is SOC, there is no specified limit to the number of lesions that can be treated with palliative local treatments (such as non-ablative external beam irradiation) in the control arm. Indications for non-study prescribed radiotherapy will always be discussed first with the sponsor and all such treatments will be carefully documented.

## Supplementary Information

**Additional file 1.**

**Additional file 2.**

## Data Availability

The data generated in this study are not publicly available. They are however available from the corresponding author upon reasonable request and with permission of the Principal Investigator and Ghent University Hospital.

## Abbreviations

4D: 4-Dimensional
AE: Adverse Event
AAPM: American Association of Physicists in Medicine
CB: Cone Beam
CD8+: Cluster of Differentiation 8 positive
CHEERS: CHEckpoint inhibition in combination with an immunoboost of External body Radiotherapy in Solid tumors
CNS: Central Nervous System
CPI: Checkpoint Inhibitor
CT: Computed Tomography
CTCAE: Common Toxicity Criteria for Adverse Events
DNA: Deoxyribonucleic Acid
EDTA: Ethylenediaminetetraacetic Acid
FFPE: Fresh-Frozen Paraffin-Embedded
GTV: Gross Tumor Volume
Gy: Gray
HIV: Human Immunodeficiency Virus
IMRT: Intensity-Modulated Radiotherapy
iRECIST: Immune Response Evaluation Criteria in Solid Tumors
mm: Millimeter
MV: Megavolt
OAR: Organ At Risk
OS: Overall Survival
PD-(L)1: Programmed Death protein-(ligand) 1
PET: Positron Emission Tomography
PFS: Progression-Free Survival
PRV: Planning organ at Risk Volume
PSA: Prostate-Specific Antigen
PTV: Planning Target Volume
PTV_optim: Optimized Planning Target Volume
QOL: Quality Of Life
RECIST: Response Evaluation Criteria in Solid Tumors
RNA: Ribonucleic Acid
SAE: Serious Adverse Event
SBRT: Stereotactic Body Radiotherapy
SOC: Standard Of Care
TIL: Tumor-Infiltrating Lymphocytes
