# Supplementary material for: Checkpoint inhibition in combination with an immunoboost of external beam radiotherapy in solid tumors (CHEERS): study protocol for a phase 2, open-label, randomized controlled trial
Source: BMC Cancer. 2021 May 7;21:514. doi: 10.1186/s12885-021-08088-w (PMC8106163; doi:10.1186/s12885-021-08088-w)
Supplement: Supplementary file 1 — Additional file 1. [file 12885_2021_8088_MOESM1_ESM.docx]

| **Data category** | **Information** |
| --- | --- |
| Trial Registry and Identifying Number | ClinicalTrials.gov  NCT03511391 |
| Date of Registration | April 17, 2018 |
| Secondary Identifying Numbers | EC2017/1678, BC-1444, CTOR17089UZG, AZSLA1905 |
| Source(s) of Monetary or Material Support | - Kom Op Tegen Kanker - Varian Medical Systems |
| Primary Sponsor | University Hospital Ghent |
| Contact for Public/Scientific Queries | *PO* MD PhD, +32 9 332 30 15, [piet.ost@uzgent.be](mailto:piet.ost@uzgent.be), Corneel Heymanslaan 10, B-9000 Ghent, Belgium |
| Public/Scientific Title | CHEckpoint Inhibition in Combination With an Immunoboost of External Beam Radiotherapy in Solid Tumors |
| Countries of Recruitment | Belgium |
| Health Condition(s) or Problem(s) Studied | Urothelial carcinoma, melanoma, renal cell carcinoma, non-small cell lung cancer, head and neck cancer |
| Intervention(s) | - Drug: atezolizumab, pembrolizumab or nivolumab per national standard of care - Radiation: Stereotactic body radiotherapy is administered to maximally 3 lesions in 3 fractions of 8 Gy concurrently with checkpoint inhibitor treatment |
| Key Inclusion and Exclusion Criteria | Inclusion: Histologically confirmed diagnosis of a solid tumor;  at least 1 extracranial tumor lesion eligible for radiotherapy; treatment with checkpoint inhibitor per standard of care.  Exclusion: Ineligible for radiotherapy; prior treatment with an anti-PD-(L)1 antibody; known additional malignancy; uncontrolled central nervous system metastases at baseline;  systemic treatment with immunosuppressive medication; diagnosis of immunodeficiency, human immunodeficiency virus (HIV), Hepatitis B or Hepatitis C infection |
| Study Type | Interventional, randomized, open-label, phase 2 |
| Target Sample Size | 98 |
| Recruitment Status | Recruiting |
| Primary Outcome(s) | Progression-free survival |
| Key Secondary Outcomes | Overall survival, tumor response as per RECIST and iRECIST, incidence of treatment-related adverse events, quality of life |

# Protocol version

Issue date: August 30, 2020

Protocol amendment number: 05, version 6

Authors: MS, NS, EH, PO

Revision chronology:

| February 14, 2018 | Original |
| --- | --- |
| April 12, 2018 | Amendment No.1, version 2: timing of radiotherapy changed based on results from phase 1 trial, sample size calculation revised, participating center added |
| July 26, 2018 | Amendment No.2, version 3: inclusion criteria expanded to allow nivolumab q4w and atezolizumab in UC, informed consent form translated to French and English, participating center added |
| December 12, 2018 | Amendment No.3, version 4: changes based on feedback of Ethical committee Jules Bordet Institute, inclusion and exclusion criteria revised |
| August 14, 2019 | Amendment No.4, version 5: informed consent form revised adding consent for retrieval of remnant tumor tissue, participating center added |
| August 31, 2020 | Amendment No.5, version 6: inclusion criteria expanded, informed consent form revised |

Important protocol modifications will be added to the trial registration form (ClinicalTrials.gov)
