# Supplementary material for: Checkpoint inhibition in combination with an immunoboost of external beam radiotherapy in solid tumors (CHEERS): study protocol for a phase 2, open-label, randomized controlled trial
Source: BMC Cancer. 2021 May 7;21:514. doi: 10.1186/s12885-021-08088-w (PMC8106163; doi:10.1186/s12885-021-08088-w)
Supplement: Supplementary file 2 — Additional file 2. [file 12885_2021_8088_MOESM2_ESM.docx]

**Institutional Review Boards (IRB)**

| **IRB name** | **Local PI** | **Address** |
| --- | --- | --- |
| Commissie voor medische ethiek  *(Central IRB)* | Prof. Dr. P. Ost | Ghent University Hospital, C. Heymanslaan 10, 9000 Ghent, Belgium |
| Commissie Medische Ethiek GZA | Prof. Dr. P. Dirix | GZA, Oosterveldlaan 24,  2610 Wilrijk, Belgium |
| Ethisch Comité | Dr. D. De Maeseneer | AZ Sint-Lucas Brugge, Sint-Lucaslaan 29, 8310 Brugge, Belgium |
| Comité Éthique | Prof. Dr. D. Van Gestel | Institut Jules Bordet, 121 Boulevard de Waterloo, 1000 Brussels, Belgium |
| Commissie medische ethiek | Dr. V. Renard | Algemeen Ziekenhuis Sint-Lucas & Volkskliniek, Groenebriel 1, 9000 Gent, Belgium |
